# Computational Modelling and Analysis of Ligand Interactions With the Dopamine Transporter

**DOI:** 10.1192/bjo.2026.11944

**Published:** 2026-06-30

**Authors:** Cassidy Keen, Michelle A. Sahai

**Affiliations:** 1 Brunel University of London, Uxbridge, United Kingdom; 2 Brunel University London, Uxbridge, United Kingdom

## Abstract

**Aims:**

This study aimed to computationally investigate interactions between DAT and a range of ligands across different conformational states using molecular docking and structural modelling, with the goal of identifying residues associated with ligand binding and potential conformational specificity.

**Methods:**

Recent Cryo EM structures of DAT representing inward-facing and outward-facing conformations were obtained from the Protein Data Bank and prepared using MODELLER.Eight compounds (Amphetamine, Buproprion, Methamphetamine, Modafinil, Methylphenidate, Mephedrone, Vanoxerine and Nortriptyline) were categorised based on binding preferences. Molecular docking was performed using Cresset Flare, generating ten poses per compound, which were ranked using Lead Finder scores. The lowest-energy protein-ligand interactions were visualised with VMD and further analysed using Schrodinger Maestro and Nanome VR.

**Results:**

In the inward-facing conformation, conserved interacting residues across compounds included Val328, Tyr156, Phe326, and Val152. In the outward-facing conformation, conserved residues included Tyr156, Val152, Ala423, Asp79, Phe320, Phe76, Phe326, Ser149, and Ser422. Asp79 and Val328 were observed to participate in interactions that differed between conformational states.

**Conclusion:**

These results identify recurring residues involved in ligand interactions with DAT and suggest conformation-dependent interaction patterns that may be relevant to transporter modulation. Future work will build on these docked complexes using molecular dynamics simulations with AI-guided analysis of protein dynamics to support drug discovery efforts aimed at the development of novel treatments for stimulant use disorder.

